# Computational Evidence for a Competitive Thalamocortical Model of Spikes and Spindle Activity in Rolandic Epilepsy

**DOI:** 10.3389/fncom.2021.680549

**Published:** 2021-06-18

**Authors:** Qiang Li, M. Brandon Westover, Rui Zhang, Catherine J. Chu

**Affiliations:** ^1^Medical Big Data Research Center, Northwest University, Xi'an, China; ^2^Department of Neurology, Massachusetts General Hospital and Harvard Medical School, Boston, MA, United States

**Keywords:** BECTS, benign epilepsy with centrotemporal spikes, CECTS, childhood epilepsy, neural mass model, electroencephalogram, Costa neural mass model

## Abstract

Rolandic epilepsy (RE) is the most common idiopathic focal childhood epilepsy syndrome, characterized by sleep-activated epileptiform spikes and seizures and cognitive deficits in school age children. Recent evidence suggests that this disease may be caused by disruptions to the Rolandic thalamocortical circuit, resulting in both an abundance of epileptiform spikes and a paucity of sleep spindles in the Rolandic cortex during non-rapid eye movement sleep (NREM); electrographic features linked to seizures and cognitive symptoms, respectively. The neuronal mechanisms that support the competitive shared thalamocortical circuitry between pathological epileptiform spikes and physiological sleep spindles are not well-understood. In this study we introduce a computational thalamocortical model for the sleep-activated epileptiform spikes observed in RE. The cellular and neuronal circuits of this model incorporate recent experimental observations in RE, and replicate the electrophysiological features of RE. Using this model, we demonstrate that: (1) epileptiform spikes can be triggered and promoted by either a reduced NMDA current or h-type current; and (2) changes in inhibitory transmission in the thalamic reticular nucleus mediates an antagonistic dynamic between epileptiform spikes and spindles. This work provides the first computational model that both recapitulates electrophysiological features and provides a mechanistic explanation for the thalamocortical switch between the pathological and physiological electrophysiological rhythms observed during NREM sleep in this common epileptic encephalopathy.

## 1. Introduction

Rolandic epilepsy (RE) is the most common idiopathic focal childhood epilepsy, characterized by a transient period of spontaneous seizures and cognitive deficits in school age children. Clinically RE is now recognized as a mild epileptic encephalopathy, characterized by the emergence of seizures and cognitive deficits during school age years (Lee et al., 2017; Ross et al., 2020). Electrographically, RE is characterized by distinctive high-voltage spikes in the Rolandic region. Both the seizures and epileptiform spikes in RE are most prominent during non-rapid eye movement (NREM) sleep (Pavlou et al., 2012). Although the genetic, electrophysiologic, and neurological features of RE have been studied in detail (Kellaway, 2000; Archer et al., 2003; Lemke et al., 2013; Mirandola et al., 2013; Lal et al., 2014; Li et al., 2017; Dryżałowski et al., 2018), the neural mechanisms underlying the stereotyped electrophysiologic features of this disease are poorly understood.

Converging evidence suggests that RE results from focal dysfunction in the Rolandic thalamocortical circuit. In support of this hypothesis, macrostructural and microstructural white matter abnormalities have been identified adjacent to the Rolandic cortex in RE (Ciumas et al., 2014; Ostrowski et al., 2019; Thorn et al., 2020). Thalamic abnormalities have also been observed in related developmental epilepsies characterized by NREM sleep-activated spikes (Fernández et al., 2012, 2017). Most recently, children with RE were reported to have focal spindle deficits in the Rolandic cortex that predicted their cognitive deficits (Kramer et al., 2021). Consistent with experimental observations in rodent models (Clementeperez et al., 2017) and cell culture (Beenhakker and Huguenard, 2009) that pathologic spikes competitively “hijack” the thalamocortical circuit that normally generates NREM sleep spindles, spindle rate anticorrelates with spike rate in RE patients (Kramer et al., 2021). As sleep spindles originate from a well-characterized thalamocortical circuit (Beenhakker and Huguenard, 2009), these findings provide a concrete pathophysiological model for RE. The potential neural mechanisms responsible for the competitive electrophysiological dynamics between the sleep activated spikes and spindles observed in RE remain unknown.

Neural computational modeling provides a tool to investigate the underlying dynamics of observed electrophysiological phenomena, test mechanistic hypotheses, and provide predictions for further experiments. Neural mass models (NMMs) describe the dynamics of a neural population through an averaged representation of specific cell types. A variety of NMMs have been developed to simulate several types of oscillatory and epileptiform patterns of brain activity in epilepsy, including high frequency oscillations, spike-wave complexes, and polyspike and wave discharges (Wendling et al., 2002; Suffczynski et al., 2004; Marten et al., 2009; Goodfellow et al., 2011; Helling et al., 2015). Similarly, NMMs have been constructed to reproduce typical rhythms during sleep, such as K-complexes, slow oscillations and spindles (Steynross et al., 2005; Cona et al., 2014; Weigenand et al., 2014; Costa et al., 2016a). Costa et al. developed a thalamocortical modeling framework to produce realistic time courses of EEG signals during NREM sleep, accurately replicating well-characterized NREM sleep architecture, including K-complexes and spindles (Costa et al., 2016b). However, for the sleep-activated epilepsies, such as RE, theoretical modeling to demonstrate the neural mechanisms underlying epileptiform spikes during NREM sleep, has not been explored.

In this study, we introduce a new neural mass model for Rolandic epilepsy (RE-NMM) incorporating the thalamocortical circuit underlying spikes and spindles in recent human and rodent studies (Clementeperez et al., 2017; Kramer et al., 2021) and genetic findings in RE (Lemke et al., 2013; Xiong and Zhou, 2017). This model extends the existing Costa model in Costa et al. (2016b) in three ways: (1) to target the dynamics of the Rolandic thalamocortical system, we include excitatory cells from the ventroposteriomedial (VPM) and ventroposteriolateral (VPL) thalamic nuclei and two types of inhibitory cells (parvocellular, PV; and somatostatin, SOM) from the reticular nucleus of the thalamus (TRN); (2) consistent with recent genetic studies in RE and idiopathic focal epilepsies (Lemke et al., 2013; Xiong and Zhou, 2017), an extra NMDA receptor-mediated current (NMDA current) is introduced in PV and SOM populations; (3) to reflect the role of ion channels mutations in idiopathic epilepsies (Poolos, 2004; Difrancesco and Difrancesco, 2015; Brennan et al., 2016), an h-type ionic current is included in the VPL and VPM populations.

We then use the model to propose the neuronal mechanisms that give rise to epileptiform spikes in NREM sleep in RE. Finally, we use the model to characterize the neuronal mechanisms responsible for the competitive dynamics observed between spikes and spindles in this disease. This work provides a new computational model to better study RE and related sleep-activated epilepsies and proposes testable hypotheses of the neuronal mechanisms underlying this common disease.

## 2. Methods

### 2.1. The Costa NMM

**Figure 1:**
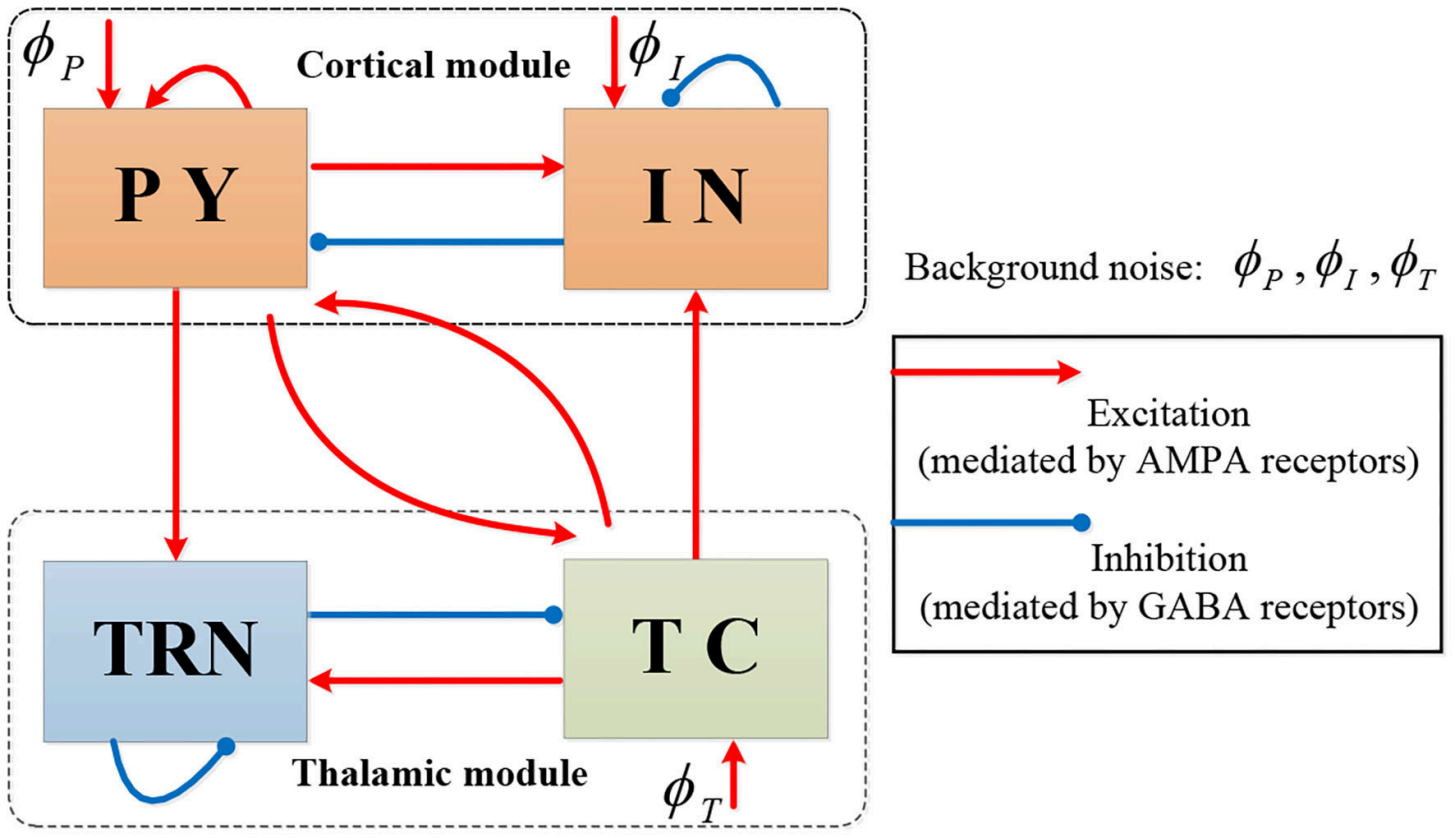
The topological structure of Costa model.

We start with the thalamocortical model in Costa et al. (2016b) which contains four neural populations: excitatory pyramidal cells (PY) and inhibitory interneurons (IN) in the cortical module, and excitatory thalamocortical cells (TC) and inhibitory thalamic reticular cells (TRN) in the thalamic module. The model's topological structure is shown in Figure 1.

In each population the dynamical evolution of neural activity is formulated by two computational operators: a sigmoid operator and a convolution operator. The sigmoid operator transforms the average membrane potential *V*(*t*) into the average firing rate *Q*(*t*), that is (Jansen et al., 1993),

(1) $$\begin{array}{l} Q\left(t\right)=\frac{Q^{max}}{1+e^{-\left(V\left(t\right)-\theta \right)\;/\sigma }}, \end{array}$$

where *Q*^*max*^, θ, σ represent the maximal firing rate, the firing threshold, and the neural gain, respectively. The convolution operator maps the firing rate *Q*(*t*) into the proportion of open synaptic channels *r*_ξ_(*t*). The membrane potential *V*(*t*) is then given by

(2) $$\begin{array}{l} \tau \dot{V\left(t\right)}=-I_{L}\left(t\right)-\underset{\xi }{\sum }I_{\xi }\left(t\right)\\ \;=-{\bar{g}}_{L}\cdot \left(V\left(t\right)-E_{L}\right)-\underset{\xi }{\sum }{\bar{g}}_{\xi }r_{\xi }\cdot \left(V\left(t\right)-E_{\xi }\right). \end{array}$$

In (2), the proportion of open synaptic channels is given by

(3) $$\begin{array}{l} r_{\xi }\left(t\right)=h_{\xi }\left(t\right)⊗\left(N\cdot Q\left(t\right)\right), \end{array}$$

where *h*_ξ_(*t*) is the impulse response function, denoted by

(4) $$\begin{array}{l} h_{\xi }\left(t\right)=\gamma_{\xi }^{2}\cdot t\cdot exp^{-\gamma_{\xi }t},t\geq 0. \end{array}$$

**Figure 2:**
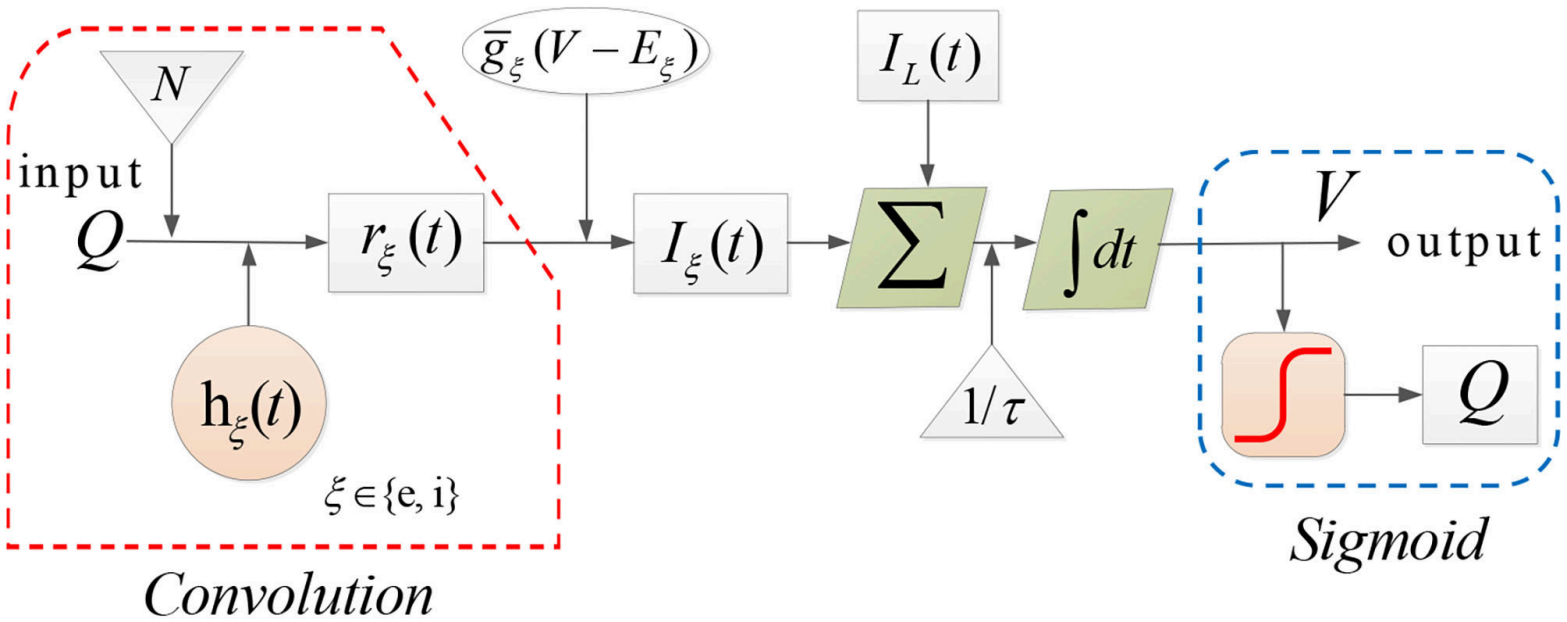
The dynamical evolution in each population in Costa model.

Here, ξ ∈ {*e, i*} denotes the type of synapse with *e* standing for AMPAergic synapses type for excitation and *i* for GABAergic type for inhibition. In Equations (2)–(4), *E*_*L*_ and *E*_ξ_ represent the reversal potential of the leak current and synaptic current, respectively, *g*_*L*_ is the maximal conductivity of the leak current conductance, ${\bar{g}}_{\xi }$ stands for the synaptic input rate that scales *r*_ξ_, τ is the membrane time constant, *N* denotes the connectivity constant, and γ_ξ_ is the rate constant of synaptic response. The dynamics of each population can be illustrated as in Figure 2.

Note that several types of currents play important roles in the Costa model. Specifically, *I*_*L*_ and *I*_ξ_ in Equation (2) represent the leak and synaptic current, respectively. In addition, several intrinsic currents are also incorporated into the calculation of *V*(*t*), including the sodium dependent potassium current *I*_*KNa*_, potassium leak current *I*_*LK*_, h-type current *I*_*h*_ and T-type calcium current *I*_*T*_.

### 2.2. Building the Rolandic NMM

#### 2.2.1. Rolandic Thalamocortical Neural Circuit

Somatosensory thalamocortical neurons from the ventrobasal (VB) thalamus process sensory information delivered between the periphery and the cortex, and also modulate thalamocortical states including sleep and seizures (Kramer et al., 2021). The VB consists of the ventral posteromedial nucleus (VPM) and the ventral posterolateral nucleus (VPL) (Jones, 2007). VPL neurons relay somatosensory, proprioceptive, and nociceptive information from the body to the somatosensory cortex (Francis et al., 2008). The VPM transmits similar information for the face (Iavarone et al., 2019). Neurons in VPL and VPM receive glutamatergic input from the somatosensory cortex, and inhibitory input from the thalamic reticular nucleus (TRN).

The TRN serves as a gate for information flow between the cortex and thalamus, which is important for supporting sleep regulation and seizure disruption. The TRN is entirely composed of GABAergic neurons and is the main source of inhibition for the VB (Gentet and Ulrich, 2003). The TRN conversely receives excitatory input from relay cells of VB, as well as from pyramidal cortical neurons. The TRN includes primarily parvalbumin-expressing neurons (PV) (Csillik et al., 2005), but somatostatin-positive neurons (SOM) have also been reported (Ahrens et al., 2015). Recently, PV and SOM were shown to coexist in the human TRN and have distinct electrophysiological properties, segregate into different anatomical locations, and participate in predominantly non-overlapping anatomical pathways that distinctly modulate somatosensory thalamocortical circuits both *in vitro* and *in vivo* (Clementeperez et al., 2017).

#### 2.2.2. Rolandic Thalamocortical Cellular Circuit

Recent evidence supports a genetic basis for Rolandic epilepsy (Lemke et al., 2013; Lal et al., 2014; Xiong and Zhou, 2017), with alterations of the gene encoding for α2 subunit of NMDARs (GRIN2A) as a major genetic risk factor for RE and related severe epileptic encephalopathies. The NMDAR is a glutamate-bound excitatory receptor with important roles in the discharge activity of neurons (Paoletti, 2011). Mutations in GRIN2A are thought to affect the NMDAR properties, including *Mg*^2+^ block levels and channel gating kinetics, giving rise to sleep-activated epileptic encephalopathies, including RE, continuous spike and wave of sleep with encephalopathy, and Landau Kleffner syndrome (Lemke et al., 2013; Reif et al., 2017; Xu and Luo, 2018). In addition, numerous studies have also suggested that other idiopathic epilepsies may be linked to a variety of ion channel mutations, which can lead to a change in the corresponding ionic current, such as the h-type current (Poolos, 2004; Difrancesco and Difrancesco, 2015; Brennan et al., 2016).

#### 2.2.3. A Neural Mass Model of Rolandic Epilepsy (RE-NMM)

**Figure 3:**
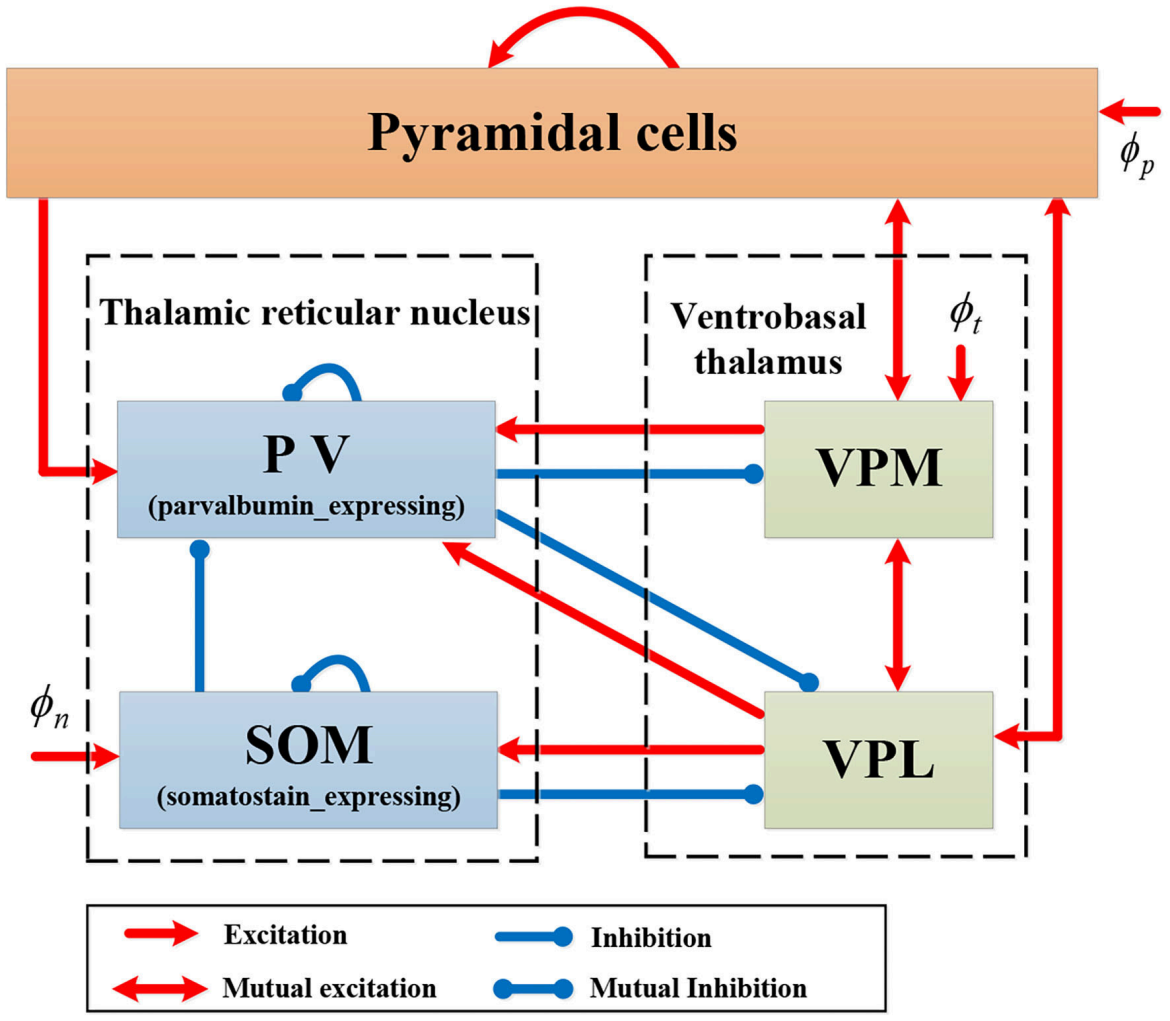
Schematic representation of model RE-NMM.

We introduce a new computational model RE-NMM based on the Costa NMM updated with features to reflect the somatosensory thalamocortical circuit and recent genetic findings in RE. First, we modify the Costa NMM to replicate features of RE by adding reticular nucleus cells and ventrobasal thalamus cells, populations present in that Rolandic thalamocortical circuit and implicated in the generation of spikes and spindles. These consist of one population in the cortical module (pyramidal cells, PY) and four populations in the thalamic module (PV, SOM, VPM, and VPL). Based on known neuroanatomical circuitry, the cortical PY population sends excitatory projection to inhibitory thalamic PV cells in the TRN and interacts mutually with excitatory thalamic VPM and VPL. Within the thalamic module, each pair of populations among PV, VPM, and VPL have excitatory or inhibitory projections; the SOM population in the TRN sends inhibitory projections to the VPL and the PV, and receives excitatory input from the VPL (Clementeperez et al., 2017). In addition, there are self-connections within PY, PV, and SOM populations, and external inputs to PY, SOM, and VPM. Note that the excitatory projections are mediated by AMPA or NMDA receptors, and inhibitory projections are mediated by GABA receptors in our model. The topological structure of our proposed RE-NMM is illustrated in Figure 3.

Motivated by the discovery that the excitatory NMDA receptors contribute to RE, we next add an additional NMDA current (*I*_*NMDA*_) involving two inhibitory populations PV and SOM in thalamus, formulated by (Zador et al., 1990; Li and Cleland, 2017).

(5) $$\begin{array}{l} I_{NMDA}=g_{N}\cdot r\cdot B\left(V_{k}\left(t\right)\right)\cdot \left(V_{k}\left(t\right)-E_{N}\right). \end{array}$$

Here, *k* ∈ {*PV, SOM*}, *V*_*k*_ is the membrane potential of PV or SOM. The function *B*(*V*) implements the *Mg*^2+^ block for the NMDA current, denoted by

(6) $$\begin{array}{l} B\left(V\right)=\frac{1}{1+\left[{Mg}^{2+}\right]e^{\left(-\mu \;\cdot 0.062\cdot V\right)}/3.57}. \end{array}$$

To characterize the effects of GRIN2A mutations on NMDA receptor, a new parameter μ is added in the expression of *B*(*V*).

Finally, the h-type current in ventrobasal thalamus neurons is a potential regulation factor for spike activities. Therefore, both VPM and VPL are equipped with the h-type current *I*_*h*_, as described in Destexhe et al. (1996)

(7) $$\begin{array}{l} I_{h}=g_{h}\cdot \left(V_{t}-E_{h}\right)\cdot \left(m_{h1}\left(t\right)+g_{inc}\cdot m_{h2}\left(t\right)\right), \end{array}$$

where *t* ∈ {*VPM, VPL*}, *V*_*t*_ is the membrane potential of VPL or VPM. The value of the conductance *g*_*h*_ is assumed to be affected by ion channel mutations. *E*_*h*_ is the reversal potential, *g*_*inc*_ denotes the conductivity scaling. The details of functions *m*_*h*1_(*t*) and *m*_*h*2_(*t*) can be found in Destexhe et al. (1993).

We model the conduction delay between the cortical and thalamic modules as a convolution with an alpha function $\tilde{h}\left(t\right)$ (Costa et al., 2016b). That is, Equation (3) is reformulated as

(8) $$\begin{array}{l} r_{\xi }\left(t\right)=h_{\xi }\left(t\right)⊗\left(N\cdot \tilde{h}\left(t\right)⊗Q\left(t\right)\right) \end{array}$$

during five transmissions “PY → PV”, “PY → VPM/VPL,” and “VPM/VPL → PY.”

Equations (9)–(29) present the full mathematical expression of the RE-NMM (see page 5–6). Note that the model output is *V*_*p*_, which is viewed as the simulated EEG signal (Hocepied et al., 2013; Costa et al., 2016b). The parameters used in RE-NMM are listed in Table 1, whose values and ranges are set based on previous studies (Zador et al., 1990; Traub et al., 1991; Destexhe et al., 1993, 1996; Costa et al., 2016b; Clementeperez et al., 2017; Li and Cleland, 2017; Iavarone et al., 2019).

(9) $$\begin{array}{l} \tau_{p}\dot{V_{p}}=-I_{L}^{p}-I_{AMPA}^{p}\left(r_{e}^{p}\right), \end{array}$$

(10) $$\begin{array}{l} \tau_{pv}\dot{V_{pv}}=-I_{L}^{pv}-I_{AMPA}^{pv}\left(r_{e}^{pv}\right)-I_{NMDA}^{pv}\left(r_{en}^{pv}\right)-I_{GABA}^{pv}\left(r_{i}^{pv}\right)\\ \;-C_{m}^{-1}\tau_{pv}\left(I_{LK}^{pv}+I_{T}^{pv}\right), \end{array}$$

(11) $$\begin{array}{l} \tau_{som}\dot{V_{som}}=-I_{L}^{som}-I_{AMPA}^{som}\left(r_{e}^{som}\right)-I_{NMDA}^{som}\left(r_{en}^{som}\right)-I_{GABA}^{som}\left(r_{i}^{som}\right)\\ \;-C_{m}^{-1}\tau_{som}\left(I_{LK}^{som}+I_{T}^{som}\right), \end{array}$$

(12) $$\begin{array}{l} \tau_{vpm}\dot{V_{vpm}}=-I_{L}^{vpm}-I_{AMPA}^{vpm}\left(r_{e}^{vpm}\right)-I_{GABA}^{vpm}\left(r_{i}^{vpm}\right)\\ \;-C_{m}^{-1}\tau_{vpm}\left(I_{LK}^{vpm}+I_{T}^{vpm}+I_{h}^{vpm}\right), \end{array}$$

(13) $$\begin{array}{l} \tau_{vpl}\dot{V_{vpl}}=-I_{L}^{vpl}-I_{AMPA}^{vpl}\left(r_{e}^{vpl}\right)-I_{GABA}^{vpl}\left(r_{i}^{vpl}\right)\\ \;-C_{m}^{-1}\tau_{vpl}\left(I_{LK}^{vpl}+I_{T}^{vpl}+I_{h}^{vpl}\right), \end{array}$$

(14) $$\begin{array}{l} I_{LK}^{\alpha }=g_{LK}\cdot \left(V_{\alpha }-E_{LK}\right),\alpha \in \left\{pv,som,vpm,vpl\right\}, \end{array}$$

(15) $$\begin{array}{l} I_{T}^{\beta }=g_{T}^{\beta }m^{2}h\cdot \left(V_{\beta }-E_{Ca}\right),\beta \in \left\{pv,som,vpm,vpl\right\}, \end{array}$$

(16) $$\begin{array}{l} {\ddot{r}}_{e}^{p}=\gamma_{e}^{2}\left(N_{pp}Q_{p}+N_{pt1}\eta_{t1}+N_{pt2}\eta_{t2}+\phi_{p}-r_{e}^{p}\right)\\ \;-2\gamma_{e}{ṙ}_{e}^{p}, \end{array}$$

(17) $$\begin{array}{l} {\ddot{r}}_{e}^{vpm}=\gamma_{e}^{2}\left(N_{t1p}\eta_{p}+N_{t1t2}Q_{vpl}+\phi_{t}-r_{e}^{vpm}\right)\\ \;-2\gamma_{e}{ṙ}_{e}^{vpm}, \end{array}$$

(18) $$\begin{array}{l} {\ddot{r}}_{e}^{vpl}=\gamma_{e}^{2}\left(N_{t2p}\eta_{p}+N_{t2t1}Q_{vpm}-r_{e}^{vpl}\right)-2\gamma_{e}{ṙ}_{e}^{vpl}, \end{array}$$

(19) $$\begin{array}{l} {\ddot{r}}_{e}^{pv}=\gamma_{e}^{2}\left(N_{r1p}\eta_{p}+N_{r1t1}Q_{vpm}+N_{r1t2}Q_{vpl}-r_{e}^{pv}\right)\\ \;-2\gamma_{e}{ṙ}_{e}^{pv}, \end{array}$$

(20) $$\begin{array}{l} {\ddot{r}}_{e}^{som}=\gamma_{e}^{2}\left(N_{r2t2}Q_{vpl}+\phi_{n}-r_{e}^{som}\right)-2\gamma_{e}{ṙ}_{e}^{som}, \end{array}$$

(21) $$\begin{array}{l} {\ddot{r}}_{en}^{pv}=\gamma_{g}\gamma_{n}\left(N_{r1p}\eta_{p}+N_{r1t1}Q_{vpm}+N_{r1t2}Q_{vpl}-r_{en}^{pv}\right)\\ \;-2\gamma_{n}{ṙ}_{en}^{pv}, \end{array}$$

(22) $$\begin{array}{l} {\ddot{r}}_{en}^{som}=\gamma_{g}\gamma_{n}\left(N_{r2t2}Q_{vpl}-r_{en}^{som}\right)-2\gamma_{n}{ṙ}_{en}^{som}, \end{array}$$

(23) $$\begin{array}{l} {\ddot{r}}_{i}^{vpm}=\gamma_{N}^{2}\left(N_{t1r1}Q_{pv}-r_{i}^{vpm}\right)-2\gamma_{N}{ṙ}_{i}^{vpm}, \end{array}$$

(24) $$\begin{array}{l} {\ddot{r}}_{i}^{vpl}=\gamma_{N}^{2}\left(N_{t2r1}Q_{pv}+N_{t2r2}Q_{som}-r_{i}^{vpl}\right)-2\gamma_{N}{ṙ}_{i}^{vpl}, \end{array}$$

(25) $$\begin{array}{l} {\ddot{r}}_{i}^{pv}=\gamma_{N}^{2}\left(N_{rr1}Q_{pv}+N_{r2r1}Q_{som}-r_{i}^{pv}\right)-2\gamma_{N}{ṙ}_{i}^{pv}, \end{array}$$

(26) $$\begin{array}{l} {\ddot{r}}_{i}^{som}=\gamma_{N}^{2}\left(N_{rr2}Q_{som}-r_{i}^{pv}\right)-2\gamma_{N}{ṙ}_{i}^{som}, \end{array}$$

(27) $$\begin{array}{l} {\ddot{\eta }}_{p}=v^{2}\left(Q_{p}-\eta_{p}\right)-2v{\dot{\eta }}_{p}, \end{array}$$

(28) $$\begin{array}{l} {\ddot{\eta }}_{t1}=v^{2}\left(Q_{vpm}-\eta_{t1}\right)-2v{\dot{\eta }}_{t1}, \end{array}$$

(29) $$\begin{array}{l} {\ddot{\eta }}_{t2}=v^{2}\left(Q_{vpl}-\eta_{t2}\right)-2v{\dot{\eta }}_{t2}. \end{array}$$

**Table 1:** The parameters and their values in RE-NMM.

**Symbol**	**Description**	**Value**	**Unit**
*C*_*m*_	Membrane capacitance in the HH model	1	μ*F*/*cm*^2^
$Q_{p}^{max}$	Maximal firing rate	30·10^−3^	ms
$Q_{y}^{max}$, *y*∈{*r*1, *r*2, *t*1, *t*2}		400·10^−3^	
**μ**	*Mg*^2+^ block factor	0.1−3	*mM*
θ_*y*_, *y* ∈ {*r*1, *r*2, *t*1, *t*2}	Firing threshold	−58.5	*mV*
σ_*p*_	Inverse neural gain	4.7	*mV*
σ_*y*_, *y* ∈ {*r*1, *r*2, *t*1, *t*2}		6	
γ_*e*_	Synaptic rate constant	70·10^−3^	*ms*^−1^
γ_*g*_		100·10^−3^	
γ_*n*_		30·10^−3^	
γ_*N*_		100·10^−3^	
*N*_*pp*_	Connectivity constant	70	/
*N*_*pt*1_, *N*_*pt*2_		3, 2	
*N*_*t*1*p*_, *N*_*t*1*t*2_		3, 1	
*N*_*r*1*p*_, *N*_*r*1*r*2_, *N*_*r*1*t*1_, *N*_*r*1*t*2_		2.6, 2.1, 4, 3	
*N*_*rr*_, *N*_*rr*2_		15, 25	
*N*_*r*2*t*2_		1.5	
*N*_*t*2*p*_, *N*_*t*2*t*1_, ***N*_*t*2*r*1_, *N*_*t*2*r*2_**		2.1, 1, 1−2	
***N*_*t*1*r*1_**		1−5.5	
τ_*p*_	Membrane time constant	30	*ms*
τ_*z*_, *z* ∈ {*r, t*}		20	
${\bar{g}}_{x\in \left\{AMPA,NMDA,GABA\right\}}$	Input rate of synaptic channel	1	*ms*
*g*_*LK*_	Conductivity of ion channel	0.042	*mS*/*cm*^2^
$g_{T}^{y}$, *y* ∈ {*r*1, *r*2, *t*1, *t*2}		1.6−2.9	
***g*_*h*_**		0.046−0.066	
*g*_*inc*_	Conductivity scaling of h-current	2	/
$E_{L}^{p}$	Nernst reversal potential	−64	*mV*
$E_{AMPA}^{p}$		0	
$E_{GABA}^{p}$		−70	
$E_{L}^{t}$		−70	
$E_{AMPA}^{t}$		0	
$E_{NMDA}^{t}$		0	
$E_{GABA}^{t}$		−70	
*E*_*LK*_		−100	
*E*_*Ca*_		120	
*E*_*h*_		−40	
ϕ_0_	Mean background noise	0	*ms*^−1^
$\phi_{p}^{s}d$	Standard deviation of cortical background noise	120·10^−3^	
$\phi_{t}^{s}d$	Standard deviation of thalamic background noise	20·10^−3^	
$\phi_{n}^{s}d$	Standard deviation of thalamic background noise	10·10^−3^	

*The subscript r_1_, r_2_, t_1_ and t_2_ indicate pv, som, vpm, and vpl respectively*.

### 2.3. Empirical Fitting of Model Parameters

To reflect the abundant spike activity common in RE, we utilized a dataset of EEGs collected from 5 children with active RE during NREM sleep (ages 4.9–14.7, 5M) with a minimum of 3 epileptiform spikes per minute during NREM sleep to constrain the parameters of the model. Clinical EEG data were referenced to the average reference. We then applied a second-order Butterworth filter with a high pass of 1 Hz, a low pass of 50 Hz, and a notch at 60 Hz to denoise RE-EEGs. To capture the focal Rolandic features, we utilized data from six centrotemporal channels: C3, C4, C5, C6, T3, and T4. Each EEG signal in the selected channels was segmented into 1-min RE-EEG epochs.

For spike detection in each RE-EEG, we applied an automated and validated spike detector, Persyst 13 (Persyst Development Corporation, San Diego) (Scheuer et al., 2017). Only epochs in which spike rates were equal to or larger than 3/min were included (*n* = 85), denoted by ***S***_***spike***_. Table 2 summarizes descriptive information about the clinical dataset.

**Table 2:** Detail information of RE-EEGs.

**Patient**	**Basic information**		**EEG dataset**					**Disease state**
	**Gender**	**Age**	**Fs (Hz)**	**No. of channels**	**Duration (s)**	**No. of spikes**	**No. of spindles**	
1	M	4.9	256	125	610	336	1,839	Active
2	M	14.7	407	69	863	287	4,567	Active
3	M	8	407	70	2,673	2,159	1,643	Active
4	M	9	407	65	494	505	1,901	Active
5	M	11.3	407	77	753	59	56	Active

We then extracted features from the EEG recordings to fit the parameters of our model. Here we calculated two features: the power spectra and the histogram of inter-spike intervals. To compute the power spectra, given an EEG signal *S* with sampling rate *F*_*s*_, the power spectral of *S* is calculated using the multitaper method (Babadi and Brown, 2014), denoted by

(30) $$\begin{array}{l} \text{P}=\left(P_{1},P_{2},\cdots \;,P_{N}\right). \end{array}$$

Here, *P*_*i*_ is the power spectral at the *i* frequency point with $N=\left[\frac{F_{s}}{2}\right]$. To compute the histogram of inter spike intervals we sorted the intervals into equal-sized bins *M*, denoted by

(31) $$\begin{array}{l} \text{D}=\left(D_{1},D_{2},\cdots \;,D_{M}\right). \end{array}$$

**Figure 4:**
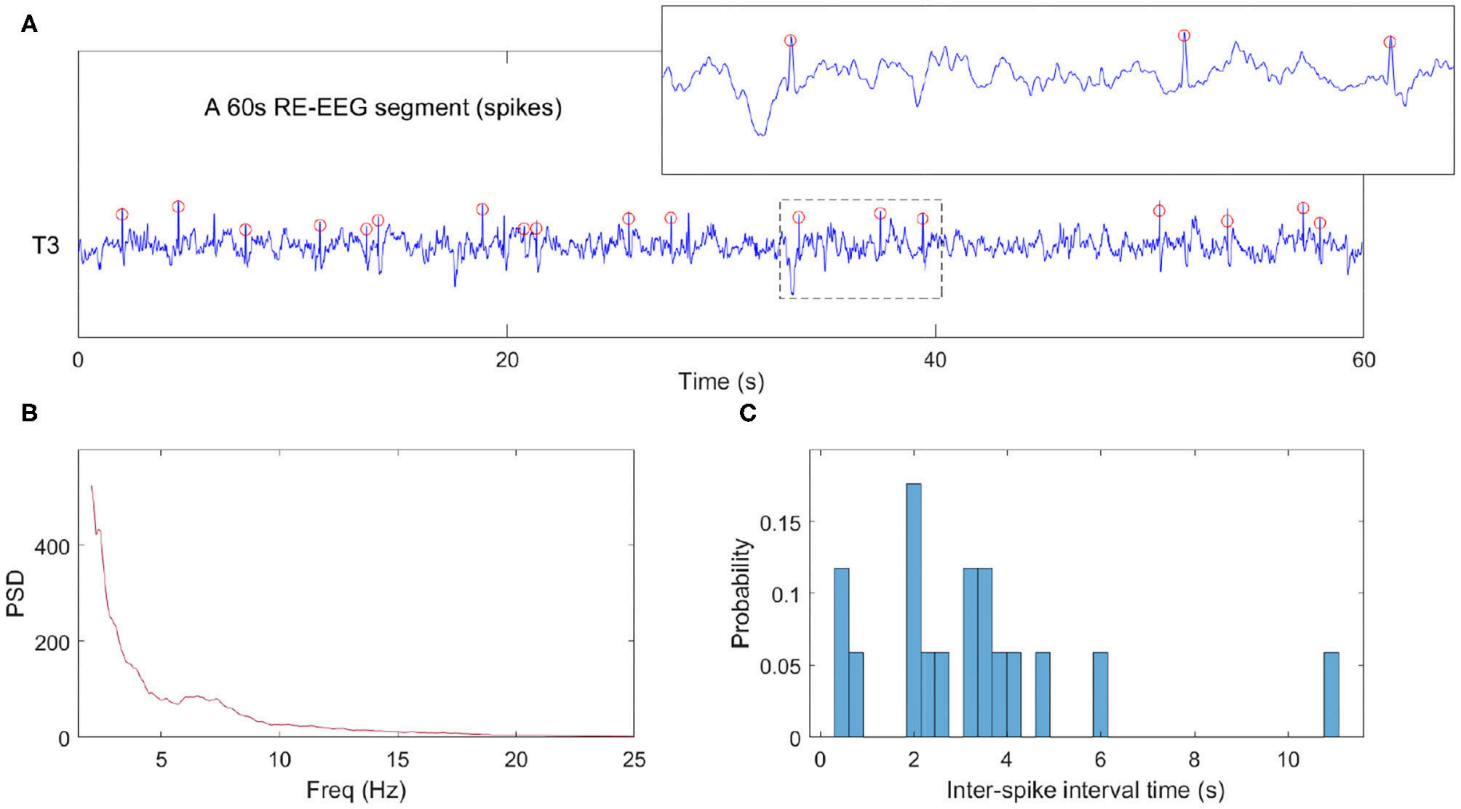
Example data analysis pipeline. **(A)** NREM sleep data segment from one clinical EEG. The red circles represent detected spikes. **(B)** The power spectra computed from a 60 s epoch. We note that in this epoch where spikes are abundant, the expected sigma bump reflecting sleep spindles is absent. **(C)** The histogram of inter-spike intervals computed from the same data epoch.

Here, *D*_*j*_ denotes the probability of inter-spike intervals in the *jth* bin. Figure 4 illustrates these data analysis steps on an example clinical EEG epoch from one subject.

Next, we estimate the posterior distribution for the parameters of our RE-NMM using the method in Hartoyo et al. (2019). Let θ be a parameter of RE-NMM and π(θ) be the prior distribution of θ, then the posterior distribution

(32) $$\begin{array}{l} p\left(\theta |\text{D},\text{P}\right)=\frac{p\left(\text{D}|\theta \right)p\left(\text{P}|\theta \right)\pi \left(\theta \right)}{p\left(\text{D},\text{P}\right)} \end{array}$$

of θ is evaluated using the Markov chain Monte Carlo (MCMC) approach. In (32), we apply the product of distributions of *P*_*i*_ to compute *p*(**P**|θ) (Thomson and Haley, 2014; Hartoyo et al., 2019), that is,

(33) $$\begin{array}{l} p\left(\text{P}|\theta \right)={\left(\frac{K^{K}e^{-K}}{\Gamma \left(K\right)}\right)}^{N}{\left(\alpha \right)}^{NK}\left(\overset{N}{\underset{i=1}{\prod }}\frac{P_{i}^{K-1}}{\overset{-}{P_{i}}{\left(\theta \right)}^{K}}\right). \end{array}$$

The likelihood function *p*(**D**|θ) is defined as

(34) $$\begin{array}{l} p\left(\text{D}|\theta \right)=\overset{H}{\underset{i=1}{\prod }}\frac{1}{\sqrt{2\pi }\sigma^{2}}e^{\frac{{\left({\overset{-}{D}}_{i}\left(\theta \right)-D_{i}\right)}^{2}}{2\sigma^{2}}} \end{array}$$

with the assumption that $\overset{-}{\text{D}}=D+\epsilon$. In this work, the error ϵ is assumed to obey the Gaussian distribution [i.e., ϵ ~ *N*(0, σ^2^)]. Here, $\overset{-}{\text{P}}$ and $\overset{-}{\text{D}}$ stands for the extracted features from the simulated EEG signals (say, the model output). A detailed explanation of each symbol in Equations (33) and (34) can be found in Moussaoui et al. (2006), Hartoyo et al. (2019).

The parameter value can be estimated by selecting the point from the Markov chain with the largest value of the posterior distribution, that is, $\hat{\theta }=\underset{\theta }{\text{arg max }}p\left(\theta |\text{D},\text{P}\right)$.

All numerical simulations are performed using MATLAB R2017b using the stochastic Runge-Kutta method of 4th order (Rosler, 2010) with step size of 0.1 ms.

## 3. Results

### 3.1. Rolandic NREM Spike Rate Is Influenced by NMDA Receptor Activity and Ionic Channel Currents

We test two parameters to generate Rolandic spikes in our model: μ and *g*_*h*_, reflecting the density of NMDA current activity and the h-type current primarily influenced by ionic channel activity. All other parameters in the model are set to their nominal values (see Table 1).

**Figure 5:**
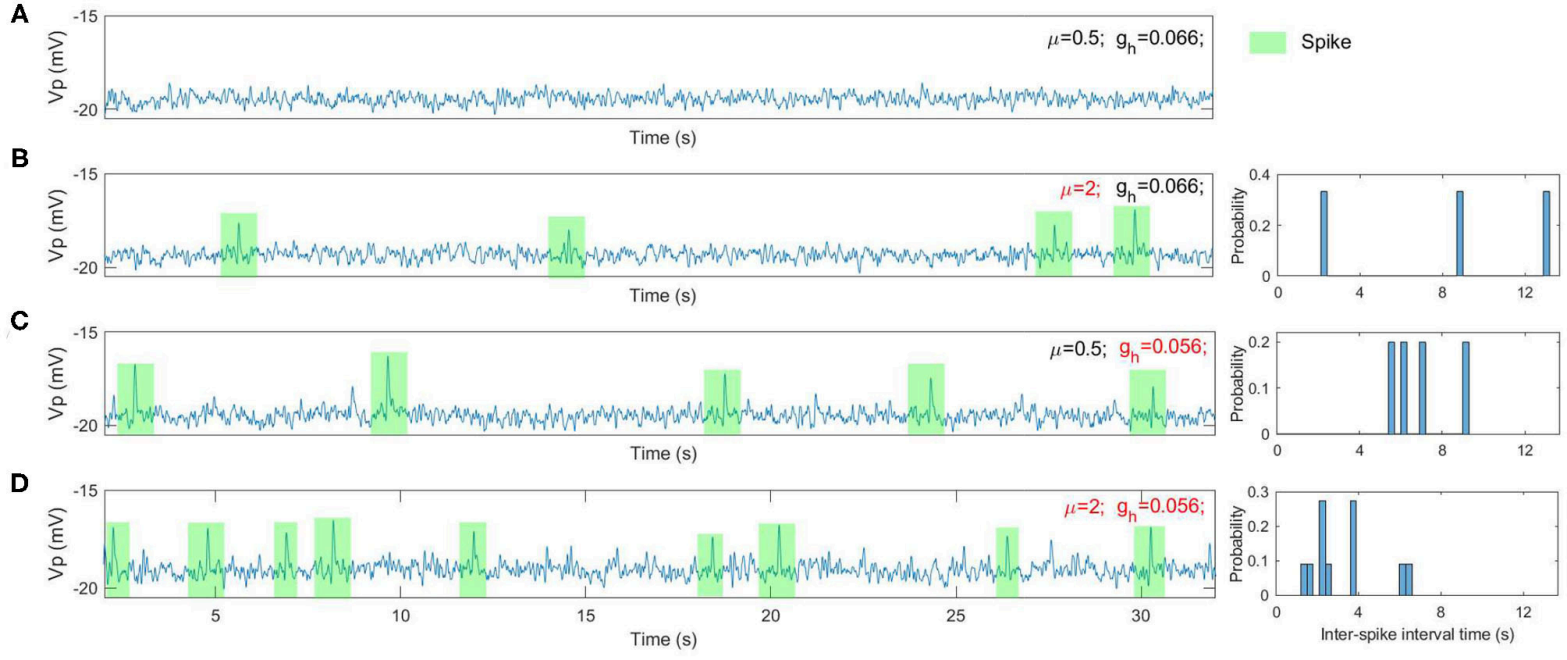
The simulated EEGs (i.e., the model output *V*_*p*_) as well as the histogram of inter-spike intervals under different situations. **(A)** μ = 0.5 and *g*_*h*_ = 0.066; **(B)** μ = 2 and *g*_*h*_ = 0.066; **(C)** μ = 0.5 and *g*_*h*_ = 0.056; **(D)** μ = 2 and *g*_*h*_ = 0.056.

We find that a range of dynamical spike activities in the RE-NMM emerge by varying the values of μ and *g*_*h*_ respectively (for example, see Figure 5). Increasing μ from 0.5 to 2 results in the emergence of spike events (see Figures 5A,B). Decreasing *g*_*h*_ from 0.066 to 0.056 also increases spike rate (Figures 5C,D). Decreasing *g*_*h*_ results in an increased spike rate (Figure 5D). The histograms of inter-spike intervals from the simulated spikes (Figures 5B–D) approximates that observed in the clinical EEGs (Figure 4C).

**Figure 6:**
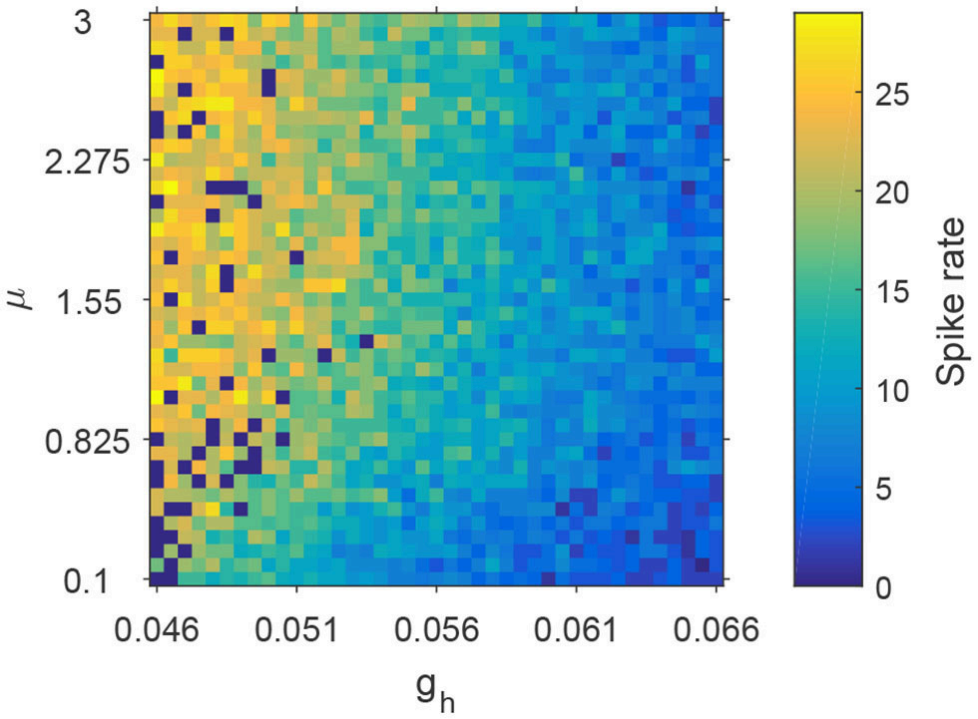
The evolution of spike rates in the parameter space μ × *g*_*h*_.

To characterize how spike rate varies with adjustments to parameters μ and *g*_*h*_, we simulated EEG signals across a range of plausible values and computed the corresponding spike rates. This allows us to observe the evolution of spikes in the parameter space of μ and *g*_*h*_. Figure 6 shows the distribution of spike rates with 41 × 41 grids in the space of μ × *g*_*h*_ ∈ [0.1, 3] × [0.046, 0.066]. We observe that higher spike rates occur for larger values of μ and smaller values of *g*_*h*_, and lower spike rates occur with smaller values of μ and larger values of *g*_*h*_. Note that larger values of μ and smaller values of *g*_*h*_ correspond to lower NMDA and h-type currents respectively (see **Figures 13A,C**). Our simulations indicate that reducing either the NMDA current or the h-type current causes an increase in epileptiform spikes.

### 3.2. Competition Between Rolandic Spikes and Spindles Is Induced by TRN Inhibition

**Figure 7:**
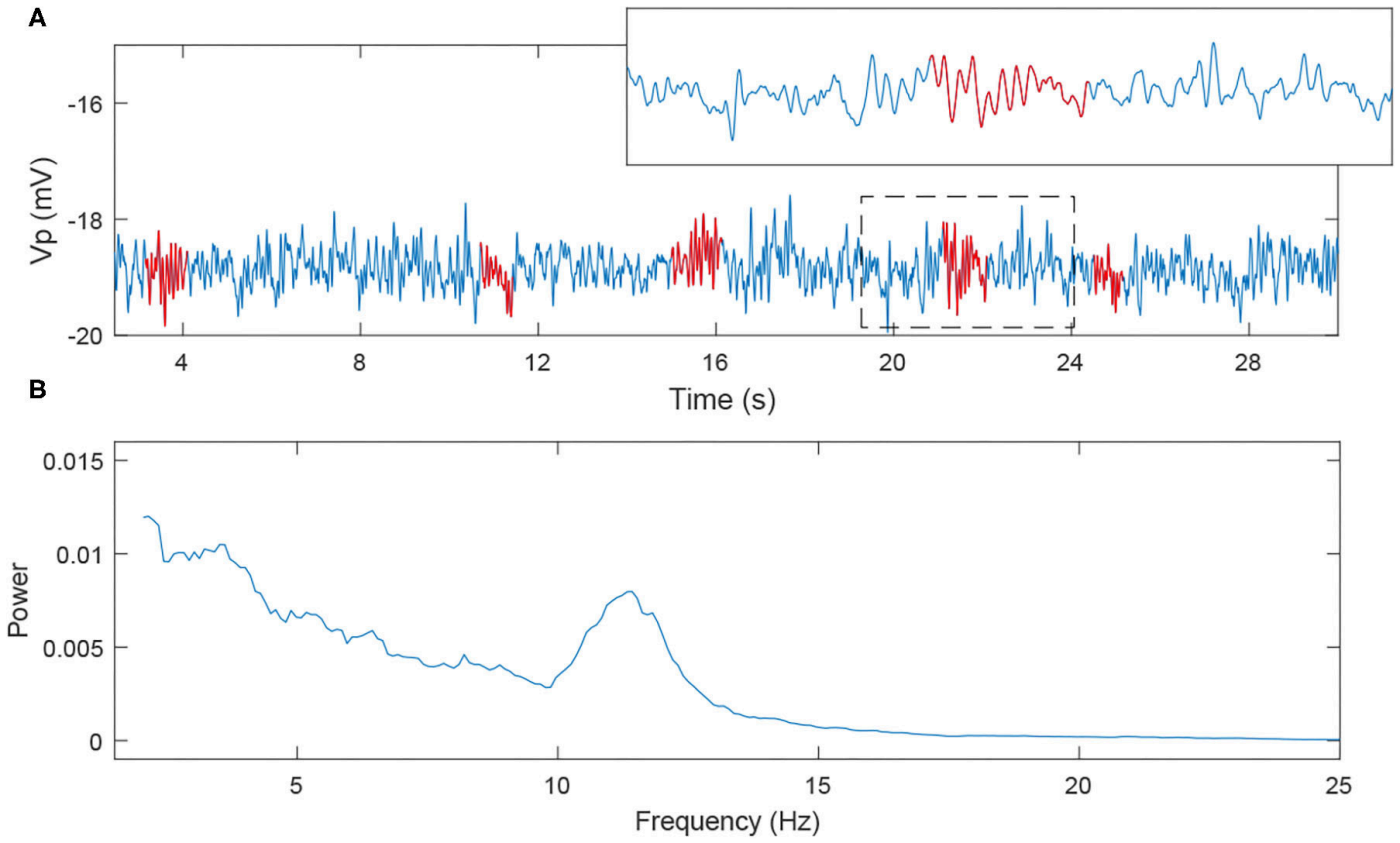
The simulated EEG signal and its power spectra. **(A)** 30s EEG segment; **(B)** power spectra (the red labeled part corresponds to the spindles).

We note that spindles can be generated by the Costa model and our RE-NMM by adjusting parameters *g*_*T*_, *g*_*h*_, and *g*_*LK*_, which are the conductance of T-type Calcium current, h-type current and potassium leak current. The dominant frequency of our simulated spindles is around 11 Hz, consistent with the sigma frequency range of human sleep spindles. An example simulated time series with a detected spindle (Wamsley et al., 2012) and accompanying power spectra is shown in Figure 7.

Based on our observations in 3.1 and previous results (Destexhe et al., 1996; Żygierewicz et al., 2001), we first fix the values of the four parameters to be μ = 2, *g*_*h*_ = 0.058, *g*_*T*_ = 2.9, and *g*_*LK*_ = 0.042, to generate spikes and spindle oscillations simultaneously in simulation signals. To test for a competitive interaction between spikes and spindles during NREM sleep in our model, we then focus on the inhibitory outputs from reticular nucleus of the thalamus, which are responsible for modulating spindles (Żygierewicz et al., 2001; Beenhakker and Huguenard, 2009). Here, we compare spike and spindle rates for three inhibitory projection strengths *N*_*t*1*r*1_, *N*_*t*2*r*1_, and *N*_*t*2*r*2_ (from population PV to VPM, PV to VPL, and SOM to VPL).

**Figure 8:**
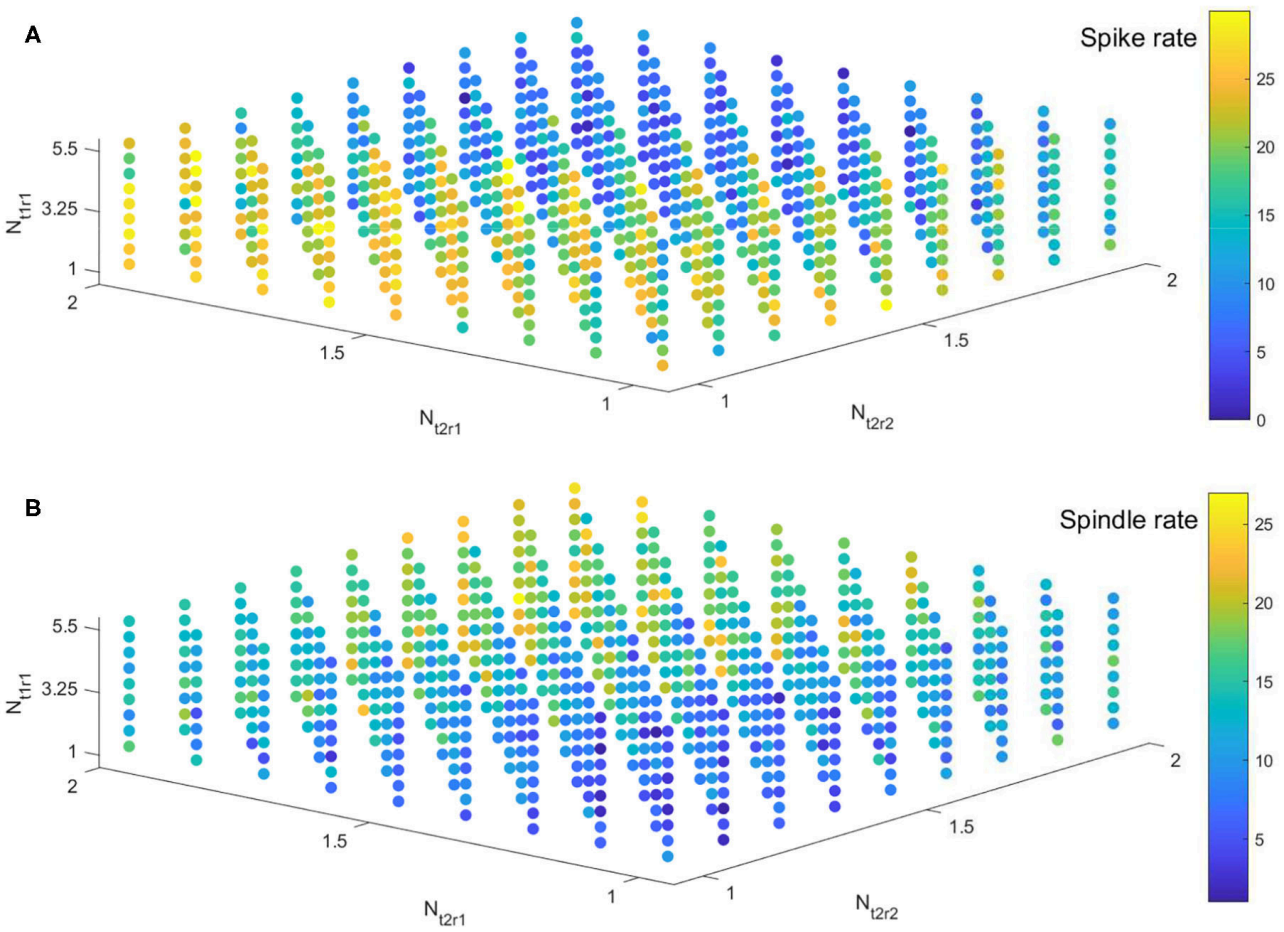
The evolution of spike rate **(A)** and spindle rate **(B)** in parameter space *N*_*t*1*r*1_ × *N*_*t*2*r*1_ × *N*_*t*2*r*2_ ∈ [1, 5.5] × [1, 2] × [1, 2].

**Figure 9:**
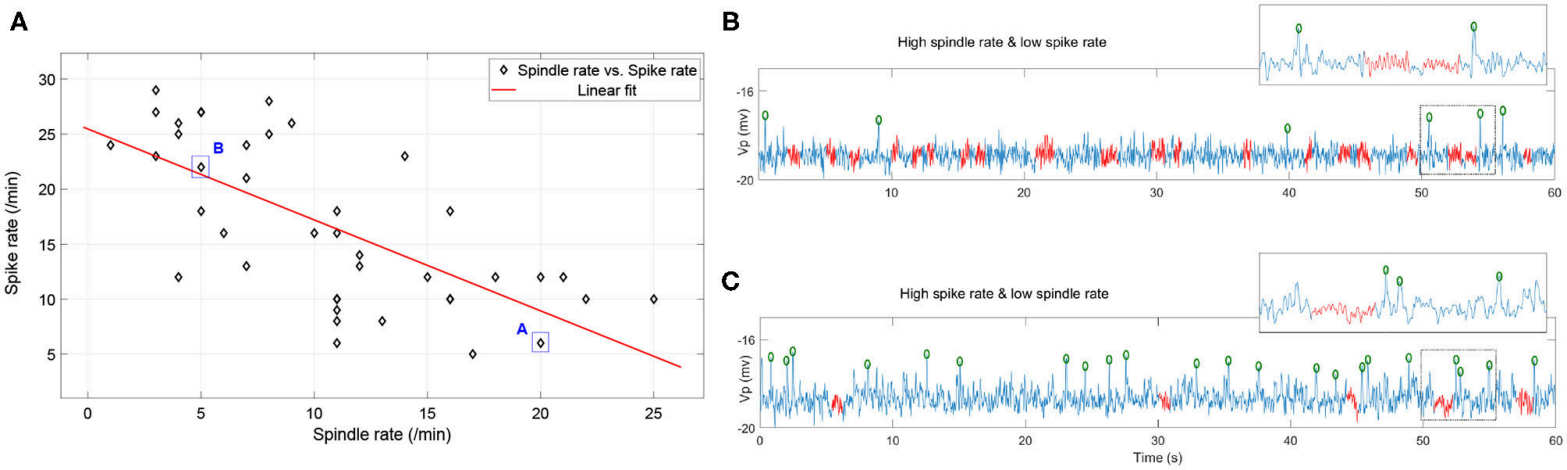
**(A)** The combinations of spike rate and spindle rate in the diagonal grids of the parameter space *N*_*t*1*r*1_ × *N*_*t*2*r*1_ × *N*_*t*2*r*2_ ∈ [1, 5.5] × [1, 2] × [1, 2]. **(B)** 60s simulated EEG segment with high spindle rate and low spike rate (corresponding to the point A); **(C)** 60s simulated EEG segment with high spike rate and low spindle rate (corresponding to the point B) (the red labeled part represents the spindles, the green circle represents the spikes).

We implement the simulation in parameter space *N*_*t*1*r*1_ × *N*_*t*2*r*1_ × *N*_*t*2*r*2_ ∈ [1, 5.5] × [1, 2] × [1, 2] with 41 × 41 × 41 grids. For ease of visualization, spike rates and spindle rates are shown for every fifth of the 41 points in Figure 8. We find that the spike rates decrease (increase) as the inhibitory projection becomes stronger (weaker), while there is the opposite relationship for spindle rate under the same situations (Figures 8A,B). The monotonously decreasing trend of the linear fit (red line) reveals that spike rate decreases with rising spindle rate, and vice versa, namely they are anticorrelated (Figure 9). Figures 9B,C show details of two points A and B in Figure 9A (marked by the square). The obtained result shows that the competitive relationship between spikes and spindles can be induced by changes of inhibitory transmission occurring in thalamus. This is consistent with the competitive relationship observed between spikes and spindles in patients with Rolandic epilepsy (see Figure 5 in Kramer et al., 2021).

To evaluate the impact of the NMDA currents on spindle rate, we tested the μ values ranging from 0.1 to 3. We found that spindle rate remained near constant at all values (4.5 ± 2.3).

### 3.3. PV and SOM Inhibitory Thalamic Populations Differentially Modulate Spikes and Spindle Activity

**Figure 10:**
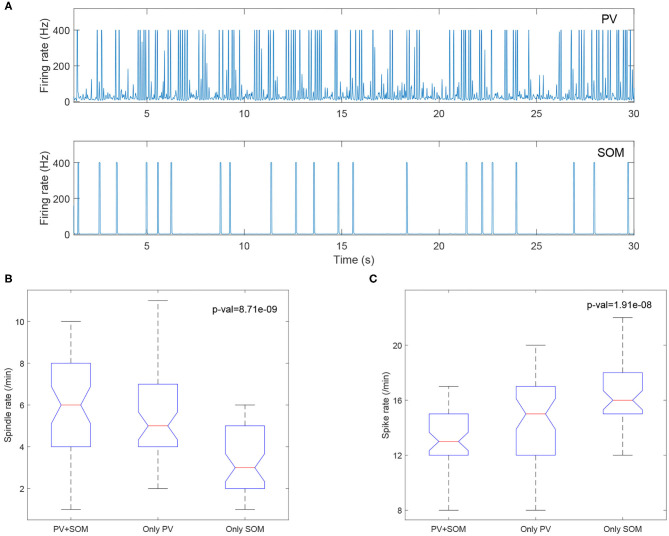
**(A)** PV cells (top) have increased firing compared to SOM cells (bottom). Boxplots of Kruskal-Wallis test statistic for the spindle rate **(B)** and spike rate **(C)** with respect to three cases (PV+SOM, only PV, only SOM).

We included both PV and SOM neurons in our thalamic inhibitory populations. These inhibitory thalamic neurons have different pre- and post-synaptic connectivity, in that the somatosensory cortex exclusively targets the PV neurons, whereas subcortical structures preferentially target the SOM cells, and PV and SOM neurons project to distinct thalamic relay nuclei. In our model, we observed that the PV neurons have much higher firing rates than the SOM neurons (Figure 10A), consistent with Clementeperez et al. (2017).

To evaluate the contributions of the PV and SOM neurons to the model, we compared the output from the original model including both PV and SOM populations, to models with only the PV population and only the SOM inhibitory thalamic populations. We found that models including only SOM neurons had lower spindle rates (Figure 10B) and higher spike rates (Figure 10C), suggesting that the PV neurons, but not the SOM neurons, preferentially support sleep-spindles and reduce epileptiform spikes.

### 3.4. Validation of Mechanisms Underlying RE Using Real RE-EEGs

**Figure 11:**
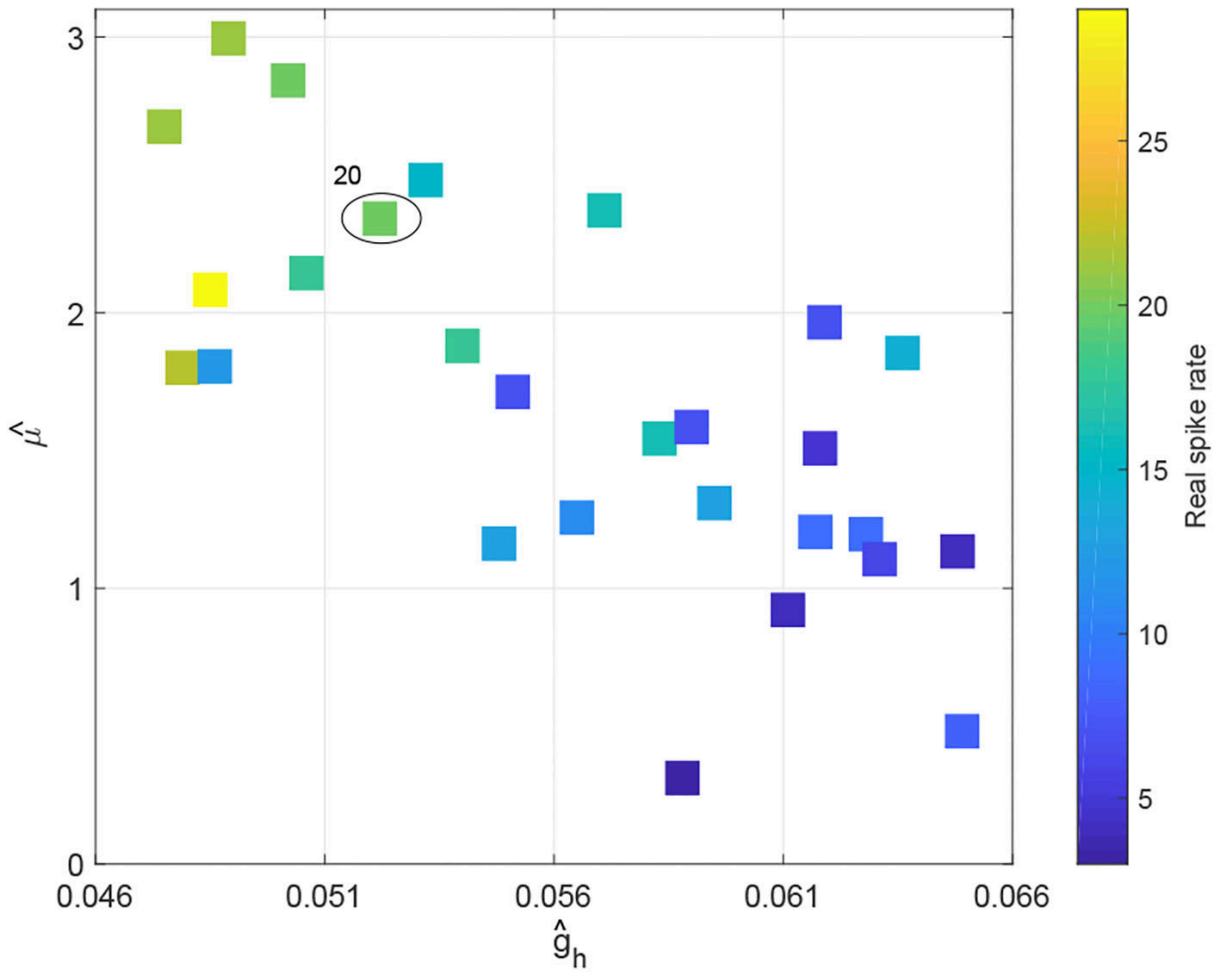
The spike rate distribution of 27 real RE-EEG segments in the estimated parameter space $\hat{\mu }\times {ĝ}_{h}$.

To observe how Rolandic spikes in real RE-EEGs evolve in the parameter space μ × *g*_*h*_, we select 27 segments from dataset ***S***_***spike***_ in which all possibilities of spike rates (from 3 to 29) are represented. Values of μ and *g*_*h*_ were estimated using an MCMC-based approach using the selected RE-EEG segments (Figure 11). We observe that RE-EEG segments with higher spike rate localize to the upper-left corner of the graph (corresponding to large $\hat{\mu }$ and small ĝ_*h*_), and RE-EEG segments at the bottom-right corner of the graph (corresponding to small $\hat{\mu }$ and large ĝ_*h*_) have relatively lower spike rates.

**Figure 12:**
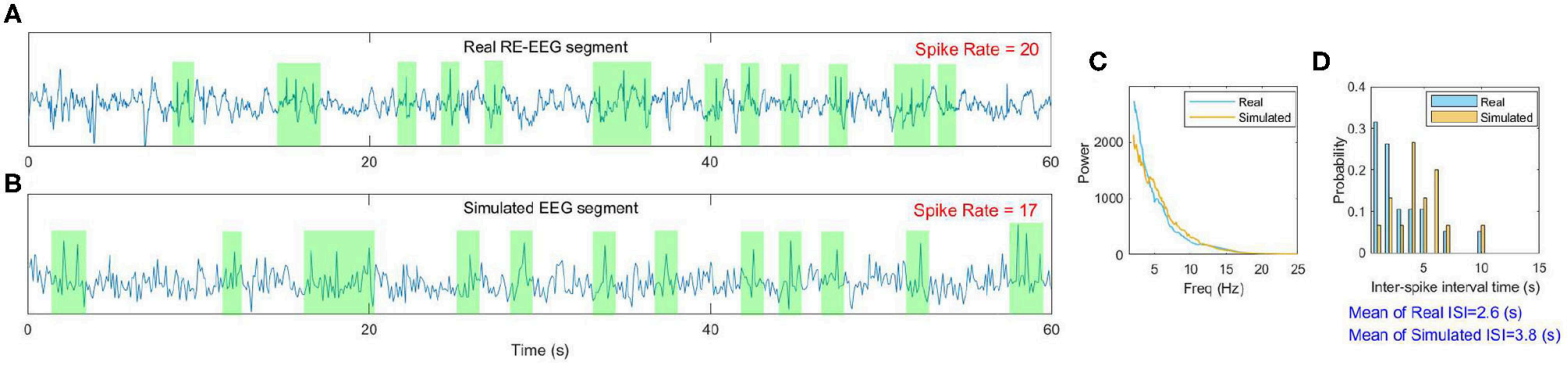
An example of the model fit to one real RE-EEG segment. **(A)** A 60s real RE-EEG segment from “patient 1”; **(B)** The simulated 60s EEG segment; **(C,D)** Comparison between the features extracted from the real EEG and the simulated one.

Furthermore, we display more details implicated in Figure 11 taking one point as an example (marked by the circle). The real RE-EEG segment at this point is shown in Figure 12A, from“patient 1”. Figure 12B illustrates the simulated RE-EEG segment, which is obtained as the model output *V*_*p*_ setting values for μ, *g*_*h*_ to their optimized values that $\hat{\mu }=2.3411$ and $\hat{g_{h}}=0.0522$ and using nominal values for other parameters. The power spectra of simulated EEG is in good agreement with that computed from the real data (Figure 12C) and the distribution of inter-spike intervals (ISI) of both simulated EEG and real one are mainly concentrated at lower inter-spike intervals, reflecting the higher spike rate in simulated EEG and real RE-EEG (Figure 12D).

**Figure 13:**
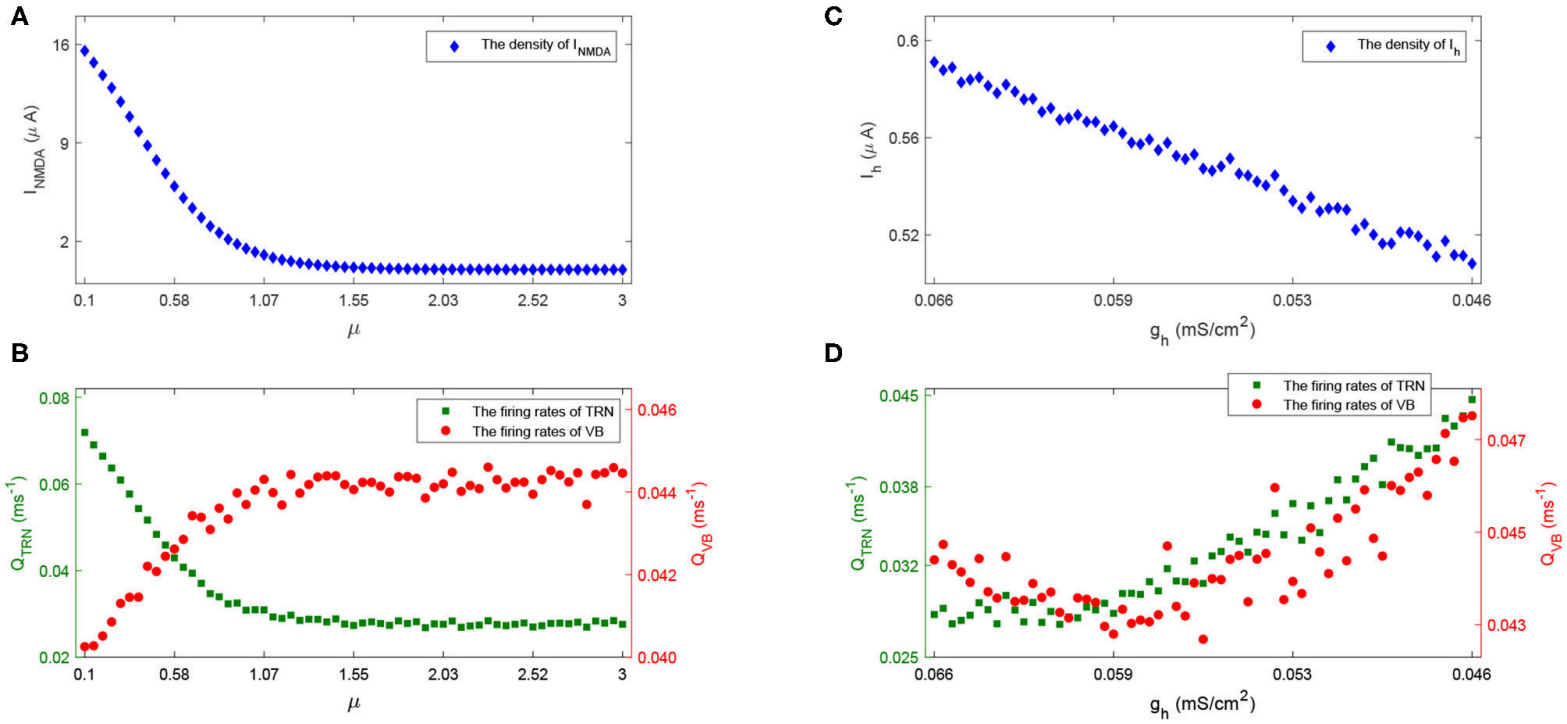
The density of *I*_*NMDA*_ **(A)** and firing rate of population TRN and VB **(B)** with the increase of μ; The density of *I*_*h*_ **(C)** and firing rate of TRN and VB **(D)** with the decrease of *g*_*h*_.

These results are plausible from a physiological point of view. Specifically, the larger the value of μ, the less the density of excitatory current *I*_*NMDA*_ (see Figure 13A), thus the weaker the inhibitory activity in the TRN (shown as the green dot in Figure 13B), the stronger the excitatory activity in the VB (shown as the red dot in Figure 13B); the reduced *I*_*h*_ (lower value of *g*_*h*_, Figure 13C) causes VB to be more excitable, which sends more excitation to TRN, and causes the TRN spike rates to increase (Figure 13D),—leading to a higher spike rate (see Figure 6). Notably, the firing rates of population VB decrease first (Figure 13D) because the reduced *I*_*h*_ could result in the increase of the time course and amplitude of after hyperpolarization potential. These observations provide new insights into how alterations in the NMDA receptor and h-type currents can result in Rolandic spikes.

## 4. Summary and Discussion

In this study we introduce a computational thalamocortical model of Rolandic epilepsy informed at both the neural circuit and cellular levels by experimental observations. We validate that the model generates spike dynamics comparable to that observed in human EEG. We then use the model to demonstrate that Rolandic spikes can be triggered and promoted by a reduced NMDA current to the inhibitory thalamic cells or h-type current in the excitatory thalamic cells; and that changes in inhibitory transmission in thalamus lead to a dynamic switch between epileptiform spikes and spindles in the shared thalamocortical circuit.

Our modeling results highlight the pacemaker role of TRN, the primary inhibitory nucleus in the thalamus, in producing spike discharges and spindles during sleep or seizure processes. Previous models- to study the spike-like activities and spindle rhythms simultaneously have primarily focused on absence epilepsy, not sleep activated syndromes. For example, Suffczynski et al. (2004) developed a thalamocortical NMM to explain the relation between mechanisms that generate spindle-like activity and those that generate spike-wave activity. Zhao and Robinson (2015) extended a thalamocortical model with bursting dynamics to explore the mechanisms underlying spike and wave seizures, as well as sleep spindles etc. Fan et al. (2017) considered a single-compartment thalamocortical model and spatially extended 3-compartment coupled network to explore the role of TRN in regulating spindles and spike-wave discharges. Knox et al. (2018) introduced a thalamocortical model to understand the mechanisms of the transformation of sleep spindles to spike and wave discharges.

Our study provides the first computational model that recapitulates thalamocortical circuit “competition” between spikes and spindles in NREM sleep. That spikes may “hijack” thalamocortical spindle circuits has been proposed in theoretical papers based on experimental observations (Beenhakker and Huguenard, 2009). We provide a mechanistic model to both test and explain these observations in RE. For example, our model suggests that decreased inhibition in the TRN may explain the onset of sleep-activated spikes and seizures in these patients. These findings are consistent with common empirical treatment approaches to use GABAergic medications to treat sleep activated spikes (Sánchez Fernández et al., 2013).

We found that reducing NMDA current and TRN output increases spike rate. In contrast, reducing TRN output decreases spindle rate, but changes to NMDA current do not impact spindle rate. Thus, changes of inhibitory outputs from the reticular nucleus can result in a competitive relationship between epileptiform spikes and sleep spindles, but changes to NMDA alone do not appear to impact this dynamic. As we tested only a subset of potential mechanisms several other potential factors, including changes to the excitatory output from thalamocortical neurons (Żygierewicz et al., 2001) or slow currents (Zhao and Robinson, 2015) could contribute to the generation of epileptiform spikes and spindles, the competitive dynamics, or their properties, such as the distribution of inter-spike intervals or the waxing-and-waning structure of the spindle oscillation.

Previous electrophysiological studies indicate that enhancing SK-channel activity promotes rhythmic bursting in TRN neurons (Wimmer et al., 2012) and the cyclical Ca-SK channel interaction may be necessary for spindle generation (Cueni et al., 2008). In contrast, we were able to produce spindles and replicate an antagonistic relationship between spikes and spindles by without including the SK-channel, but by tuning just three parameters reflecting the potassium leak current, h-type current and T-type calcium current (Destexhe et al., 1996; Costa et al., 2016b) suggesting that the SK type current is not required for these dynamics. However, SK channels may play an important role in more subtle dynamics of thalamocortical rhythms, such as bursting activity (Ritter-Makinson et al., 2019), that were not explored here.

## 5. Conclusion

Our study provides the first computational model that both recapitulates and provides a mechanistic explanation for the thalamocortical “competition” between epileptiform spikes and sleep spindles in the most common epileptic encephalopathy. These data provide hypotheses for empirical testing of the neural mechanisms underlying this disease and related sleep-activated epilepsie.

## Data Availability Statement

The original contributions presented in the study are included in the article/supplementary material, further inquiries can be directed to the corresponding author/s.

## Author Contributions

QL, MW, RZ, and CC designed and performed the research as well as wrote the paper. All authors contributed to the article and approved the submitted version.

## Conflict of Interest

The authors declare that the research was conducted in the absence of any commercial or financial relationships that could be construed as a potential conflict of interest.
